# Mitigating the Twin Threats of Climate-Driven Atlantic Hurricanes and COVID-19 Transmission

**DOI:** 10.1017/dmp.2020.243

**Published:** 2020-07-14

**Authors:** James M. Shultz, James P. Kossin, Attila Hertelendy, Fredrick Burkle, Craig Fugate, Ronald Sherman, Johnna Bakalar, Kim Berg, Alessandra Maggioni, Zelde Espinel, Duane E. Sands, Regina C. LaRocque, Renee N. Salas, Sandro Galea

**Affiliations:** Center for Disaster and Extreme Event Preparedness (DEEP Center), Department of Public Health Sciences, University of Miami Miller School of Medicine, Miami, Florida; NOAA’s National Centers for Environmental Information (NCEI), Center for Weather and Climate, Madison, WI; Department of Information Systems and Business Analytics, College of Business, Florida International University, Miami, FL; Harvard Humanitarian Initiative, Harvard University, Harvard T.H. Chan School of Public Health, Boston, MA; Woodrow Wilson International Center for Scholars, Washington, DC; Craig Fugate Consulting LLC, FEMA, Gainesville FL; FEMA (ret), River Forest, IL; Department of Public Health Sciences, University of Miami Miller School of Medicine, Miami, Florida; Johns Hopkins Bloomberg School of Public Health, Baltimore, MD; Sylvester Comprehensive Cancer Center, Department of Psychiatry and Behavioral Sciences, University of Miami Miller School of Medicine, Miami, Florida; Ministry of Health (ret), Nassau, New Providence, The Bahamas; Division of Infectious Diseases, Massachusetts General Hospital, Harvard Medical School, Boston, MA; Department of Emergency Medicine, Massachusetts General Hospital, Harvard Medical School; Center for Climate, Health, and the Global Environment, Harvard T.H. Chan School of Public Health, Harvard Global Health Institute, Boston, MA; School of Public Health, Boston University, Boston, MA

**Keywords:** climate change, climate drivers, COVID-19, evacuation, hurricane, mitigation, pandemic, sheltering

## Abstract

The co-occurrence of the 2020 Atlantic hurricane season and the ongoing coronavirus disease 2019 (COVID-19) pandemic creates complex dilemmas for protecting populations from these intersecting threats. Climate change is likely contributing to stronger, wetter, slower-moving, and more dangerous hurricanes. Climate-driven hazards underscore the imperative for timely warning, evacuation, and sheltering of storm-threatened populations – proven life-saving protective measures that gather evacuees together inside durable, enclosed spaces when a hurricane approaches. Meanwhile, the rapid acquisition of scientific knowledge regarding how COVID-19 spreads has guided mass anti-contagion strategies, including lockdowns, sheltering at home, physical distancing, donning personal protective equipment, conscientious handwashing, and hygiene practices. These life-saving strategies, credited with preventing millions of COVID-19 cases, separate and move people apart. Enforcement coupled with fear of contracting COVID-19 have motivated high levels of adherence to these stringent regulations. How will populations react when warned to shelter from an oncoming Atlantic hurricane while COVID-19 is actively circulating in the community? Emergency managers, health care providers, and public health preparedness professionals must create viable solutions to confront these potential scenarios: elevated rates of hurricane-related injury and mortality among persons who refuse to evacuate due to fear of COVID-19, and the resurgence of COVID-19 cases among hurricane evacuees who shelter together.

## INTRODUCTION

In 2020, the converging threats of climate-driven hurricanes and active coronavirus disease 2019 (COVID-19) transmission vastly complicate the process of preparing coastal and island-based populations for seasonal hurricane risks. Response is impeded by fundamental incompatibilities between the most effective population protection strategies for each of these hazards. The proven pillars of hurricane mitigation – evacuation and sheltering – bring people together, transporting them in groups and shielding them inside fortified structures.^1-5^ Strategies introduced to slow the spread of COVID-19 – physical distancing, quarantine, self-isolation, and conscientious hygiene – intentionally keep people apart.^6-10^ In search of workable solutions for protecting populations, despite conflicting approaches, we examine (1) how climate change may be making Atlantic hurricanes more dangerous; (2) how COVID-19’s ease of transmissibility complicates hurricane preparedness; (3) how evacuation and sheltering procedures may spread COVID-19; and (4) how communities can creatively manage concurrent hurricane and pandemic threats in 2020, when there is no COVID-19 vaccine.

### Climate Change Is Likely Making Atlantic Hurricanes More Hazardous to Population Health

There is mounting evidence that climate change is making hurricanes more hazardous while rendering island and coastal communities more vulnerable.^11,12^ Recent hurricane seasons have showcased how climate change is likely contributing to stronger, wetter, and slower-moving Atlantic storms as they come onshore over populated areas.^13-20^ Hurricane Dorian’s devastation of the northwest Bahamas in 2019 provides a compelling example of this trifecta of storm characteristics.^21,22^ Now, in 2020, climate scientists are forecasting an above-average Atlantic hurricane season.^23,24^ Populations at greatest risk for direct strikes from Atlantic storms are residents of small island states spread throughout the Caribbean and persons living along the east coast of the United States, Mexico, and Central America.

Hurricanes are tropical cyclones, rotating systems of thunderstorms that form over tropical or subtropical waters, have a low-level closed circulation,^25^ and predominantly move westward and poleward.^26-28^ Rotating winds are the signature hazard and the basis for categorizing hurricane strength and damage potential.^11,25^ Water hazards, including storm surge, coastal wave action, intense precipitation, and inland freshwater flooding, produce the greatest peril.^11,17,19,25^

**FIGURE 1:**
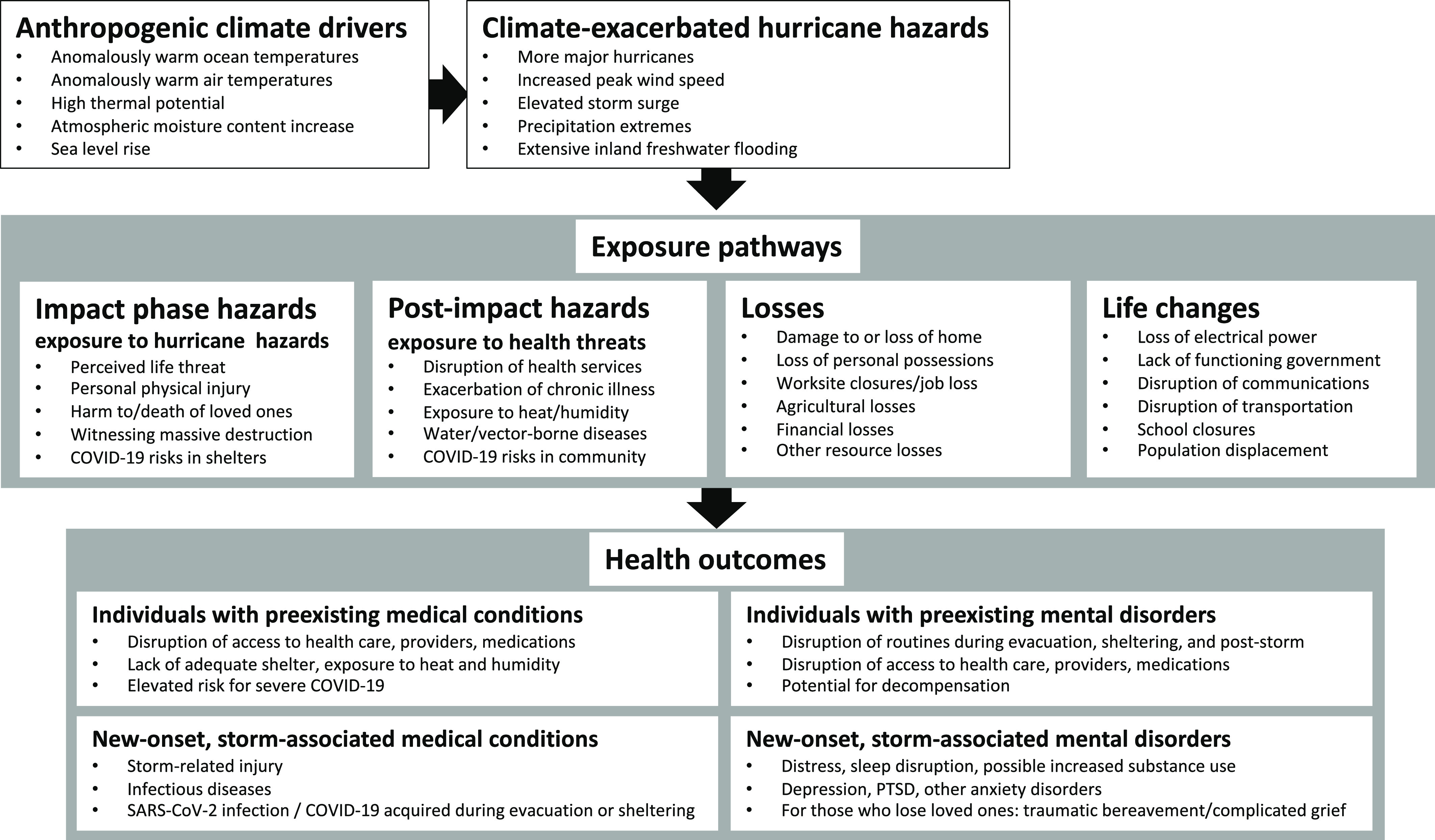
Climate-driven Atlantic Hurricanes: Exposure Pathways and Health Outcomes

Increased Atlantic hurricane activity has been observed over the past 50 years.^11,14^ Human actions, particularly the burning of fossil fuels, cause Earth to retain more heat energy than it releases, altering the climate system and shaping hurricane behavior, acting through the intermediaries of “climate drivers.”^29^ Climate drivers include anomalously warm ocean and air temperatures (7 consecutive years, 2014–2020, are the hottest on record), warm ocean waters extending far down into the depths of the sea, and increased atmospheric moisture holding capacity.^11,16-19,30^ Climate drivers amplify hurricane hazards – peak wind speeds, intensification rates, rainfall totals, and flood risks.^13,20^ Sea level rise, linked to the thermal expansion of warm ocean waters and the melting of polar ice, exacerbates hurricane damage from storm surge and coastal wave erosion. Higher seas, propelled by stronger winds, overtop coastal dwellings and severely damage the built environment. Climate-driven storms cause protracted power outages and disrupt health care services. Physical and mental health consequences and storm-related mortality may cascade for months following impact (Figure 1).^12,13,31-37^

As the climate progressively alters the behavior of Atlantic storms, upgrading measures to safeguard communities in the path has never been more urgently needed.

### COVID-19 Transmission Dynamics that Complicate Hurricane Preparedness

What distinguishes the 2020 Atlantic hurricane season is that population protection from extreme storms is on a collision course with the onward-marching COVID-19 pandemic.^38^ Despite impressive progress toward developing a safe, tolerable, immunogenic COVID-19 vaccine, population immunization will not be underway prior to the peak months of the 2020 season.^39,40^ In the absence of a vaccine, the critical mainstays of hurricane mitigation, evacuation, and sheltering – actions that move and gather people together – may inadvertently facilitate the spread of COVID-19. To better understand the infectious disease risks inherent in hurricane sheltering and evacuation, we describe what is known about the ease of transmission of COVID-19 in relation to epidemic patterning, transmission dynamics of severe acute respiratory syndrome coronavirus 2 (SARS-CoV-2; the virus that causes COVID-19), and human behaviors in community settings.

#### Epidemic Patterning

COVID-19 transmission occurred at an astonishing speed, circumnavigating the planet during the single month of March 2020 with more than 200 countries and territories reporting cases to the World Health Organization.^38^ As a novel coronavirus and emerging “spillover” zoonotic disease, the entire world population (7.8 billion persons) is susceptible to the SARS-CoV-2 infection.^41^ COVID-19 has a comparable or higher reproduction number (R_0_) than influenza; on average, each infected, and infectious, person will spread the virus to multiple other persons, leading to sustained transmission.^42,43^ One plausible scenario is that a second, potentially more virulent, COVID-19 pandemic wave will sweep the globe, beginning during the later months of the Atlantic hurricane season.^42^ Furthermore, COVID-19 transmission may diminish but will not disappear during the hot, humid peak months of the Atlantic hurricane season.^44^ Therefore, when evacuation warnings are issued and shelters are opened, COVID-19 will be viable and circulating in the communities that are taking evacuation and sheltering precautions as a hurricane approaches.

#### SARS-CoV-2 Transmission Dynamics

Airborne spread through large droplet nuclei, submicron aerosols, and turbulent gas clouds poses a great risk for rapid transmission.^45-53^ The common guidance to observe 6 feet of separation – based on large droplet nuclei generally traveling less than this distance and settling quickly – is likely inadequate, particularly in enclosed spaces like shelters. Smaller particles can be detected, suspended in air, at much larger distances.^49,52^ When sheltering – whether in homes or community shelters – COVID-19 transmission can still occur even if persons sheltering together attentively maintain 6 feet of separation. SARS-CoV-2 can also be transmitted by direct contact with persons who are shedding the virus or with contaminated high-touch surfaces or inanimate objects (fomites).^49-51^ COVID-19 spread from persons who show no symptoms (asymptomatic and presymptomatic spread) has been documented worldwide.^54-57^ Asymptomatic transmission undermines the ability to screen people using temperature and health checks at the entrance to community shelters; some persons who are symptom-free will nevertheless be infectious and capable of infecting others. SARS-CoV-2 has already undergone a spike mutation, resulting in a more transmissible strain that produces higher viral loads, rising to prominence globally.^58^

#### Community Behaviors

In the United States, nationwide easing of mitigation measures began shortly before the beginning of the 2020 Atlantic hurricane season, actions that may increase the appearance of case clusters, sporadic superspreading events,^59,60^ and possible large-scale resurgence that may extend throughout the peak months of the hurricane season.^61^ Daily case counts were already rising within the first month for states that rapidly relaxed restrictions.

**FIGURE 2:**
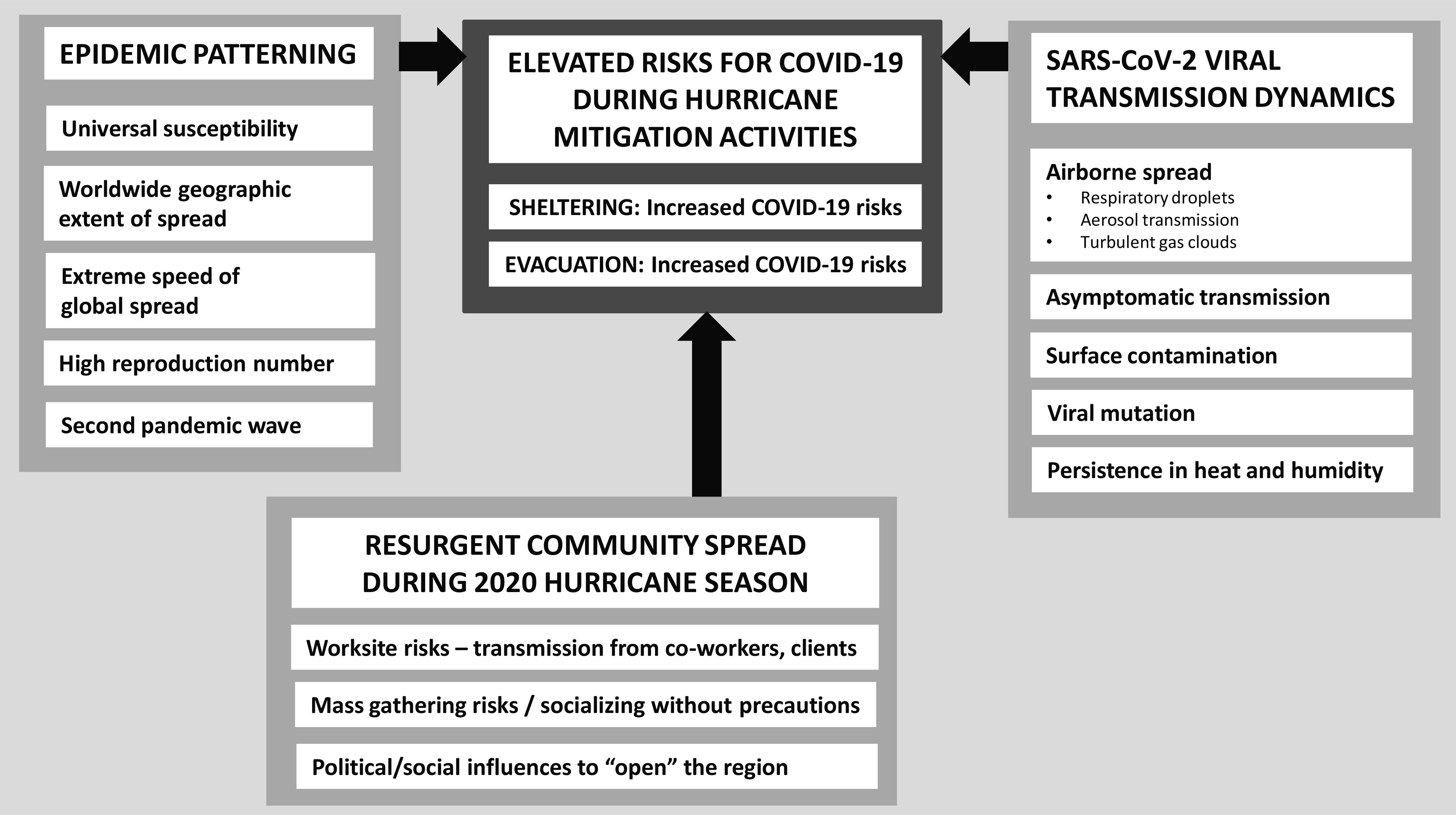
Ease of COVID-19 Transmissibility Elevates Risks for COVID-19 Transmission During Hurricane Mitigation Activities

Collectively, these epidemic patterns and SARS-CoV-2 transmission dynamics, coupled with individual and collective actions that may restimulate community transmission, elevate COVID-19 risks during hurricane evacuation and sheltering procedures (Figure 2).

## COMPLEXITIES AND INCOMPATIBILITIES: MITIGATING HURRICANE AND COVID-19 RISKS IN 2020

Mitigation strategies for Atlantic hurricanes and for COVID-19 include some commonalities but also important incompatibilities. We examine stockpiling, sheltering, and evacuation strategies.

### Stockpiling Vital Supplies

Preparing for both hurricanes and COVID-19 includes stockpiling survival supplies. Since the inception of COVID-19 stay-at-home orders in March 2020, many households have stocked up on drinking water, non-perishable foods, and paper goods; similar basics are recommended for sheltering in place during hurricanes. Supplies for COVID-19 lockdowns also prioritize soaps, bleach, alcohol disinfectants, and anti-bacterial wipes. Hurricane stockpiling features items needed for prolonged power outages: batteries, flashlights, weather radios, cell phone chargers, grills, and generators. Procuring multiple months of prescription medications, keeping gas tanks full, and having extra cash on hand are common to both. COVID-19 has made citizens more alert and attentive to disaster warnings and more attuned to media messaging.

On the downside, some high-demand essentials, including toilet paper and cleaning supplies, are chronically or cyclically out of stock. Some areas experience sporadic shortages of fresh produce and meats. In the context of the scope and scale of COVID-19, strains on the food supply and vital supplies may extend to regional, national, or even global levels. Increasing numbers of families are already dependent on government financial assistance and drive-through mass feeding programs to meet their nutritional needs. Emergency managers are planning for contingencies where, following hurricane impact, storm survivors may face acute food insecurity and join the ranks of those currently tapping into food reserves – while COVID-19 continues to spread.

### Population Sheltering Options and COVID-19 Risks

Sheltering residents in storm-resistant structures, located inland and away from coastlines, is a stalwart among hurricane mitigation strategies. Population sheltering operates in tandem with effective hurricane tracking, timely warnings, and planned evacuation of coastal zones at high risk for storm surge and high-velocity wind damage. Together, these proven strategies have decreased hurricane-related injuries and deaths. These procedures are initiated in advance of hurricane landfall throughout coastal communities and island states located in the forecast “cone” of an oncoming Atlantic storm.

#### Sheltering in Place at Home or with Family Members and Friends

Most residents whose homes are not in evacuation zones elect to shelter in place inside their own homes; for these families, COVID-19 risks would remain unchanged if the composition of the household stays the same. However, COVID-19 risks rise if the residents invite family members or friends to join them. On the flipside, many families living in evacuation zones prearrange to “double up” in the inland homes of relatives. However, in 2020, these well-tested sheltering options that gather people together in a confined space may set off household-level case clusters of COVID-19.

#### Congregate Community Shelters

Congregate sheltering involves safeguarding a large number of evacuees housed inside structurally durable community “mass care” centers operated by local emergency management with staff supplementation from county employees or American Red Cross volunteers. Traditionally, large spaces (eg, school gymnasiums) are used for most shelter residents, and, historically, each individual is allotted a 4- by 5-foot space (that may be reduced to 3- by 5-foot spacing when larger numbers are seeking refuge). Now, in the pre-vaccine era of COVID-19, these mass sheltering arrangements represent the antithesis of physical distancing. Placing local residents from diverse neighborhoods together in an enclosed space for a prolonged duration elevates risk for spreading COVID-19.^62^ As a partial solution, emergency managers are planning to open more smaller spaces within large designated shelters (eg, using individual classrooms inside schools). Fortunately, only a small proportion of citizens rely on community shelters. For example, in Miami-Dade County, Florida, with a population approaching 3 million, the shelter capacity is approximately 100 000 spaces spread across 80 schools.

#### Non-congregate Shelters

In 2020, many communities are exploring ways to expand shelter accommodations to allow smaller groups to shelter with more physical space and bathrooms (to maintain hygiene) per resident. This approach features “non-congregate sheltering,” opening locations where each individual or household has living space that offers some level of privacy (like hotels, motels, casinos, dormitories, or retreat camps).^5^

#### Individualized Sheltering Arrangements

Some families with financial means make their own shelter plans (eg, reserving hotel rooms or campground spaces, renting a recreational vehicle) that facilitate physical distancing in settings away from coastlines and the projected path of the storm.

#### Post-impact Sheltering for Persons Who Are Unable to Return Home Due to Storm Damage

A proportion of the population will be unable to return to their homes due to structural damage, flooding, immovable debris, and infrastructure disruptions.^2^ Temporary housing options will be arranged for displaced survivors; and again this raises COVID-19 contagion risks. Depending upon the geographic extent of damage, numbers of households affected, and available options for emergency housing, the Federal Emergency Management Agency (FEMA) will take an active role and may need to call in the US military for support following catastrophic storm strikes.

### COVID-19 Risks For Those Who Evacuate and Hurricane Risks For Those Who Fail to Evacuate

Historically, hurricane evacuation has successfully decreased direct exposure to storm hazards and concomitant risks for physical harm and psychological trauma. However, in 2020, evacuation raises COVID-19 risks for those who evacuate and those who host evacuees.

#### COVID-19 Risks for Those Who Evacuate

Evacuation often involves a marathon obstacle course of gridlocked highways, motor vehicle crash scenes, and fuel shortages along the route. Specific to COVID-19, the party traveling together will share the close confines of a vehicle for many hours or several days. Along the way, there may be periodic encounters with concentrated groups of strangers at freeway plazas, rest stops, restaurants, or crowded hotels. Those who opt to fly to a safe locale will experience COVID-19 risks in airport departure lounges and onboard their flights.

In 2020, evacuation will move COVID-19 along with the travelers, endangering persons at the destination point. Already in early 2020, many residents of COVID-19 epicenters in the northeastern United States migrated to second homes along the hurricane coast, particularly in south Florida; they set off clusters of COVID-19 cases when they arrived. Similarly, hurricane evacuees will almost certainly include presymptomatic persons who are infectious and may transmit COVID-19 to fellow evacuees, persons they encounter where they stop to eat and rest along the route, and those who receive them at the end of their travels.

#### Hurricane Risks for Those Who Fail to Evacuate

The high proportions of persons who do not heed evacuation orders pose a perennial problem; these persons sustain severe preventable injuries, place first responders who must rescue them at risk, and fill the surge beds at area medical centers. A primary concern in 2020 is that fear of contracting COVID-19 will cause even more residents to stay in their homes and be exposed to injurious, climate-amplified hurricane hazards.

**FIGURE 3:**
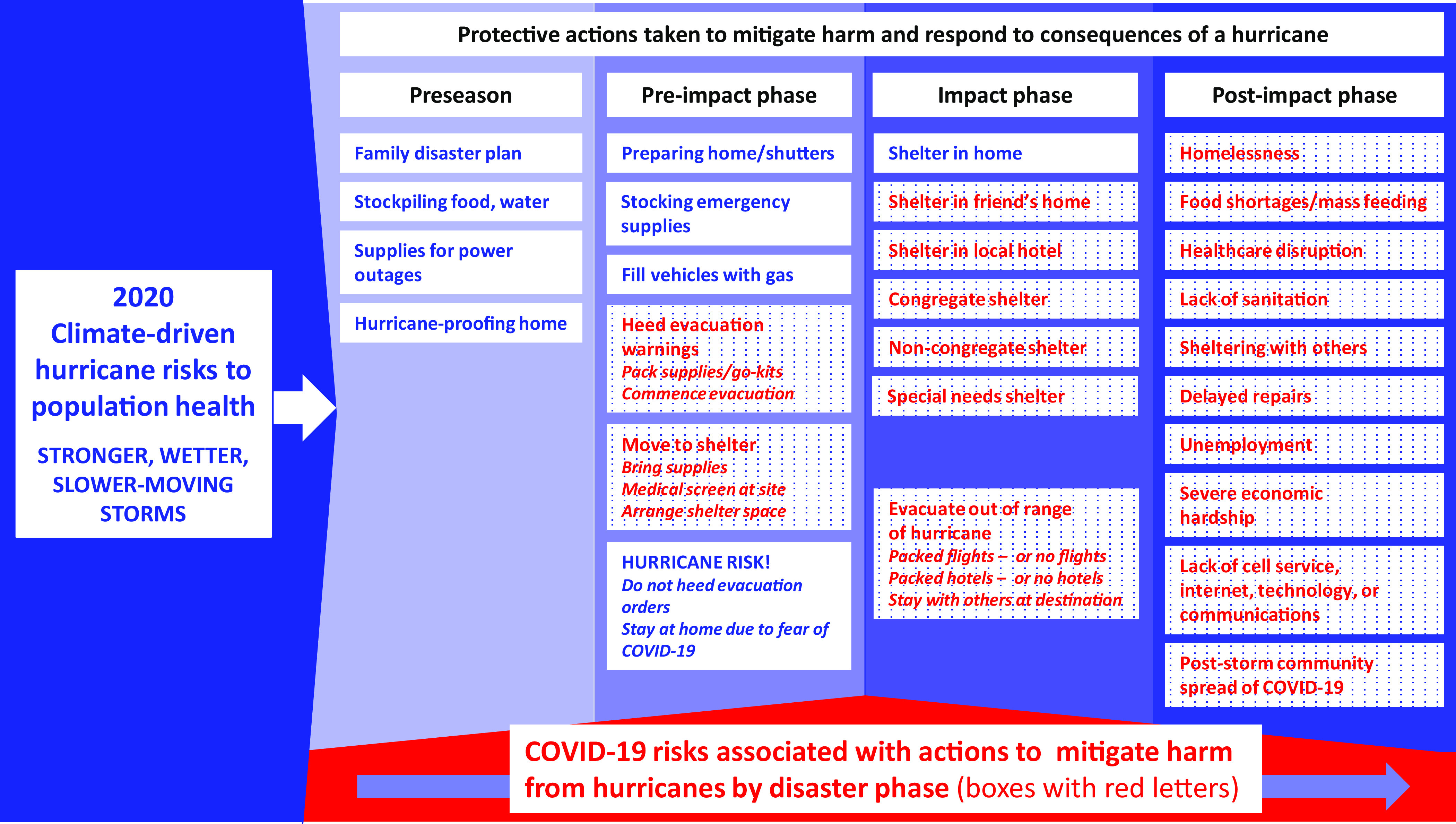
COVID-19 Risks Associated with Actions to Mitigate Harms from Hurricanes by Phase of the Disaster Cycle

Figure 3 illustrates many points of intersection along the disaster cycle where protective actions taken by households and communities to mitigate harm from hurricanes may potentiate the spread of COVID-19.

### Improvising Solutions to Concurrent Hurricane and Pandemic Threats in 2020

When considering the formidable challenges in dealing with a hurricane while COVID-19 is circulating in the community, there are no easy solutions available to neutralize both threats simultaneously and eliminate offsetting risks. However, there are ways to limit medical and psychological trauma from exposure to hurricane hazards while minimizing an uptick in COVID-19 transmission.

#### Focus on the 2020 Hurricane Season

There is a compelling need to focus energies on solutions for a twin threat scenario during the 2020 hurricane season while no COVID-19 vaccine is available. Experts predict that a safe, effective vaccine will be available for population-wide immunization in early 2021. If this prediction is borne out, the double-risk scenario may not recur during future hurricane seasons.

#### Maintain a COVID-19 Prevention Lifestyle

Research suggests that the non-pharmaceutical interventions and anti-contagion policies invoked around the globe in early 2020 have spared hundreds of millions of persons worldwide (including an estimated 60 million in the United States) from contracting COVID-19.^1,9^ During the pre-vaccine period of global vulnerability to COVID-19, continuing throughout the 2020 hurricane season and into early 2021, the greatest leverage to minimize harm from a combined hurricane-pandemic disaster scenario is to continue to observe precautions to limit the spread of COVID-19, even as steps are taken to reopen portions of society. It will be much safer to shelter or evacuate – and to elect to take these protective actions – if viral transmission in the storm-threatened community remains subdued. Along the hurricane coast, this will require continued physical distancing, avoidance of mass gatherings, continued use of masks, and careful adherence to handwashing, coupled with diligent public health surveillance (case identification, isolation, quarantine, and contact tracing).

#### Adapt Community Sheltering to COVID-19

The need to modify operational protocols at congregate shelters to reduce risks for COVID-19 transmission has received concerted attention from both the Centers for Disease Control and Prevention (CDC) and FEMA. CDC released guidance on sheltering that attempts to balance the competing risks of minimizing exposure to hurricane hazards and reducing the likelihood of COVID-19 transmission.^1^ The National Mass Care Strategy has produced documents on related themes: COVID-19 congregate sheltering, feeding procedures in congregate shelters, and non-congregate sheltering.^3-5^ CDC asserts, “Large congregate shelters should be the last resort.”^1^ CDC recommends “sheltering in place” when possible as the optimal choice. Using “non-congregate” sites, including hotels, dormitories, and small shelters accommodating fewer than 50 residents is the next most preferred choice.^1,5^ When mass care shelters must be opened, CDC recommends speedy demobilization as soon as the storm passes to minimize time spent in a shared public space. All persons who use mass shelters are advised to self-quarantine for 14 days after leaving the shelter.

CDC focuses on COVID-19 infection control in community shelter settings.^1,3^ This includes daily symptom checks (temperature, respiratory symptoms, shortness of breath). Residents with symptoms would be isolated away from others in the shelter. All residents would be required to wear masks, stay within their assigned spaces (generally 6 feet apart from others), and eat sanitized and prepackaged meals.^1,3,4^ Handwashing stations would be set up along with continuous cleansing and disinfecting of high-touch surfaces.

FEMA’s *2020 COVID-19 Pandemic Operation Guidance for the 2020 Hurricane Season (POG)* provides high-level guidance for local emergency managers to consider when dealing with the COVID-19-transformed risk landscape in their communities.^2^ The POG is replete with checklists that include sheltering considerations both during and after the storm. Infection control precautions emphasize having adequate supplies of cleansing agents and personal protective equipment. FEMA also focuses on protecting nursing home residents and special needs populations at elevated risk for COVID-19.^2^

#### Adapt In-home Sheltering to COVID-19 and Educate the Public

One solution that is overlooked and must be prioritized is to adapt and extend infection control guidance developed for congregate shelters for use by large segments of the public who will be sheltering together in homes with family and friends. Many of the same principles apply in mass shelters and in homes or smaller sheltered enclaves – space people apart, do not share food and utensils, wear masks, wash hands meticulously, check for symptoms, separate anyone who is ill. Currently, critical information on how to shelter safely with friends and family is not being communicated to the public.

#### Evacuation Behavior Is the Major Question Mark

A primary unknown for emergency managers is whether, in the era of COVID-19, residents will evacuate when warned to do so. Historically, high proportions of persons have failed to heed mandatory evacuation orders.^63-65^ Studies have examined motivations for evacuating, or refusing to evacuate, and there are few consistent findings. Evacuation was more common for younger ages, female gender, white race, and families with children.^64^ Those who were warned by a trusted source such as a city official and those who perceived higher risks for harm and damage (from stronger storms) were more likely to evacuate. Surviving previous near-miss experiences and believing that one’s home is structurally secure decrease the likelihood of evacuation. Importantly, in the studies reviewed, only 1 hazard was in play (eg, hurricane, wildfire, flood) and evacuation moved people away from that hazard to safety. In 2020, with twin competing threats, evacuation transports people away from the ravages of a hurricane but potentially into situations that raise risks for COVID-19. How persons will weigh these competing risks is not well understood.

Framing and communicating the risks will be critical. The public needs to know that (1) Atlantic hurricanes have never been more hazardous, (2) responding to evacuation orders is essential for family safety, and (3) sheltering options can be made safer by taking precautions to minimize the spread of COVID-19. Former FEMA Administrator, Craig Fugate, has alerted the nation to the reality that “emergency communications will be the next challenge of COVID-19.”^66^ An individual’s risk perception informs their decision-making, especially in the context of profound uncertainty as COVID-19 risks interweave with protective options to safeguard families from hurricane hazards. This means trusted information from reliable sources is even more essential. Fugate’s advice for responding to evacuation orders in 2020 is simple, concise, and precise (Box 1).

BOX 1 **Craig Fugate (FEMA Administrator 2009–2017):**

**Evacuation Guidance for the 2020 Atlantic Hurricane Season**

• **Standard evacuation message is even more applicable for dealing with COVID-19.**
• **COVID-19 or no COVID-19, if you live in a hurricane evacuation zone, plan now to leave if a storm is threatening and you are ordered to evacuate.**
• **If you live in a hurricane evacuation zone:**
  • Move inland to a safer location.
  • For US coastal communities, evacuate 10s of miles rather than 100s of miles.
  • Know where you will go.
  • Evacuate to a shelter with family or friends outside of the evacuation zone.
  • Other options: hotels or motels.
  • Consider temporary facilities activated by the US Army Corp of Engineers as COVID-19 alternative care sites.
  • As the last resort evacuate to an emergency public shelter – and take your pets with you.
• **If you live outside of the evacuation zone:**
  • If safe, staying home may be your best option.
  • Prepare for extended power outages.
  • Prepare for disruptions to communications (keep a portable radio in your disaster kit).
• **Fewer people evacuating reduces the risk of COVID-19 spread and allows those who must evacuate to use available hotels and motels.**

BOX 2 **Frederick Burkle, Jr., MD, MPH, DTM, FAAP, FACEP:**

**COVID-19, Climate Change, Hurricanes, and Complex Threat Scenarios in Context**

*Critical masses of evidence indicate that the frequency, duration, and intensity of extreme events affecting populations are on the rise. These are attributable to a number of converging megatrends defined as global, sustained, and often slow to form forces that will define our future. The increasing numbers of sudden-onset natural disasters on every continent; rapid and unsustainable urbanization plagued by insufficient public health infrastructure; scarcity of water, food, and energy; and deforestation and loss of biodiversity systems that serve as the biological oxygen of the world and the major safeguard against infectious diseases exemplify the megatrends of climate extremes. Furthermore, in the last 5 decades, over 30 new diseases have emerged, as well as a resurgence of old diseases and multiple-drug-resistant microorganisms. The former distinction between naturally occurring disasters and man-made crises is becoming less relevant in light of current multifaceted crises*.

#### Apply Lessons from Infectious Disease Outbreaks in Shelters During Previous Hurricanes

For years, hurricane shelters have physically isolated persons who arrive with evident illness symptoms and “reverse-isolated” those who are immunosuppressed or are otherwise susceptible to infectious disease. Several states along the hurricane coast have a special system in place to identify, register, transport, and shelter persons who have special medical needs, particularly those who are electronically dependent. Many of these patients have chronic lower respiratory disease and require oxygen concentrators. These shelters have auxiliary power generators and are staffed by public health nurses. Infection control measures that have been successful in special needs shelters can be adapted for congregate mass care settings.

The literature on infectious disease outbreaks in shelters is scant. However, lessons can be drawn from the rare outbreak involving 20 cases of influenza A (H3) in a densely populated Texas “megashelter,” housing more than 3300 survivors from 2017 Hurricane Harvey for 4 weeks.^67^ Authors described their “holistic surveillance and response” model, coordination among onsite agencies, real-time epidemiologic surveillance, and enhanced isolation and hygiene practices to limit spread. One major distinction: influenza vaccine was available and used to immunize other residents.

#### Learn from Each 2020 Storm and Continuously Refine Protocols

Predictions call for multiple landfalling 2020 storms. Starting with Tropical Storm Cristobal that came ashore along the US Gulf Coast on June 8, it is important to rapidly generate and share after-action reports that examine shelter operations and mandated evacuations. Lessons gleaned early in the season can refine and shape evacuation and sheltering protocols to improve mitigation procedures when stronger storms threaten and strike later in the season.

#### Placing COVID-19, Climate Change, and Synchronous Threats in Context

Dr. Frederick Burkle, Jr., shared his keen observations, providing context for the current COVID-19 pandemic, the ongoing climate crisis, and derivative combined threat scenarios that may increasingly become the new normal (Box 2).^68,69^

## CONCLUSION

We have now documented (1) how climate change influences the hazard properties of Atlantic hurricanes, making them more dangerous; (2) what we are learning about the transmission of COVID-19 that must be integrated into plans for hurricane mitigation; (3) how various options for evacuation and sheltering influence the spread of COVID-19; and (4) how communities can manage concurrent threats of climate-driven Atlantic hurricanes superimposed on COVID-19 transmission risks. It is imperative to forthrightly provide timely, multidisciplinary scientific, and pragmatic information about both risks – specific to the storm and specific to the current status of COVID-19 in the imperiled community – to guide individuals, their households, their communities, and decision-makers to select the best options to protect life and emerge resilient.
